# Fight against COVID-19: The case of antiviral surfaces

**DOI:** 10.1063/5.0043009

**Published:** 2021-03-01

**Authors:** Kamyar Shirvanimoghaddam, Mohammad Karbalaei Akbari, Ram Yadav, Adil K. Al-Tamimi, Minoo Naebe

**Affiliations:** 1 Carbon Nexus, Institute for Frontier Materials, Deakin University, Geelong, Australia; 2 Faculty of Science, Department of Solid State Sciences, Ghent University, Ghent, Belgium; 3 Civil Engineering Department, American University of Sharjah, Sharjah, United Arab Emirates

## Abstract

The COVID-19 pandemic is the largest global public health outbreak in the 21st century so far. Based on World Health Organization reports, the main source of SARS-CoV-2 infection is transmission of droplets released when an infected person coughs, sneezes, or exhales. Viral particles can remain in the air and on the surfaces for a long time. These droplets are too heavy to float in air and rapidly fall down onto the surfaces. To minimize the risk of the infection, entire surrounding environment should be disinfected or neutralized regularly. Development of the antiviral coating for the surface of objects that are frequently used by the public could be a practical route to prevent the spread of the viral particles and inactivation of the transmission of the viruses. In this short review, the design of the antiviral coating to combat the spread of different viruses has been discussed and the technological attempts for minimizing the coronavirus outbreak have been highlighted.

## I. INTRODUCTION AND BACKGROUND

**FIG. 1.**
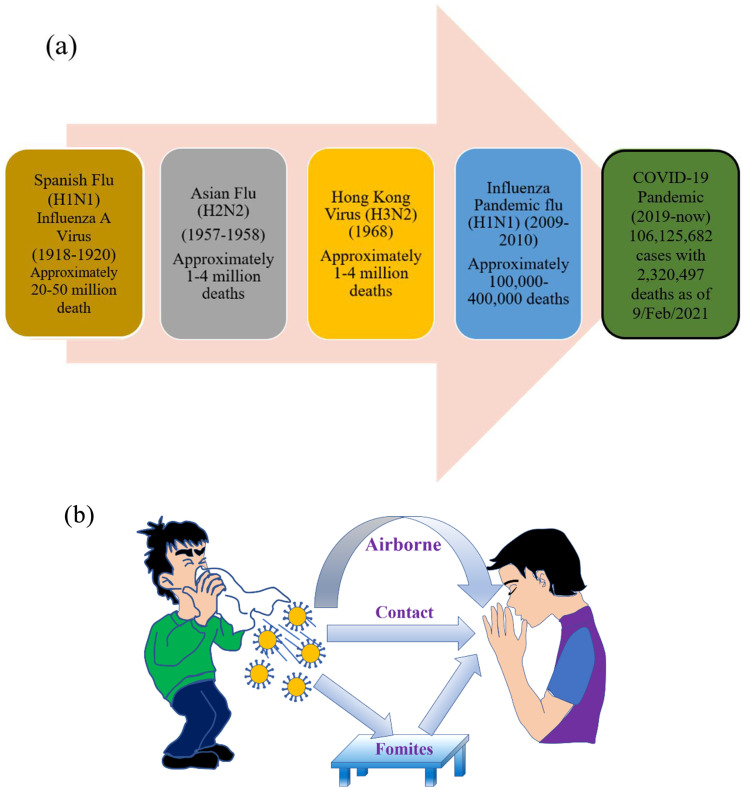
(a) Chronological description of some recent global pandemics [data obtained from the World Health Organization (WHO)].^2^ (b) Schematic representation of the mode of spread from an infected person to a healthy person.

The world has encountered a number of viral pandemics in recent history that have caused tremendous morbidity and fatality, as shown in Fig. 1(a).^1,2^ Viruses do not possess the ability to reproduce independently; rather, they need a living host cell, and further replication into more virions requires the viruses to first attach to or absorb onto a host cell followed by penetration, synthesis, maturation (assembly and packaging into new virions), and release of mature virions.^3^ Human interaction with viruses may involve direct or indirect pathways.^4^ For instance, viral infections such as SARS, MERS, and recently COVID-19 are related to directly or indirectly contacted respiratory disease.^4^ These respiratory diseases are transported by the infected person through coughing, sneezing, or even talking, as delineated in Fig. 1(b).^5^ In some conditions, virion-laden respiratory droplets deposit and dry onto various objects and further transmit to humans via touching the contaminated surface by hand or other means. Such contaminated surfaces are called “fomites” and form the major source for the spread of the virus and morbidity among people and communities. Stephens *et al.* defined fomites as any inanimate object, and when contaminated, they serve as a means to transfer infectious agents to a new host.^6^ Therefore, fomites are not limited and can be extended to different surfaces, such as mobile phones, high touch public places, hospital equipment and facilities, clinical materials and consumables, and surface of packaged materials and food. There are some disadvantages of the current protocols of disinfecting contaminated surfaces using bleaches or similar chemicals. The current protocols are expensive processes as they should be repeated frequently, they release irritant gases, and, most probably, it is not safe to dispose contaminated plastics and containers after use. Common surfaces are used regularly by the public and therefore can be repeatedly contaminated following each use, and certain disinfectants can also trigger asthma and can be linked to other chronic respiratory conditions.^7^ Therefore, there is a need to think about the large scale use of self-cleaning surfaces in our everyday life. Since most of the available antiviral surfaces are working temporarily, the focus is on the development of the surfaces with long-term stability and durability.

## II. DESIGN OF ANTIVIRAL COATINGS

**FIG. 2.**
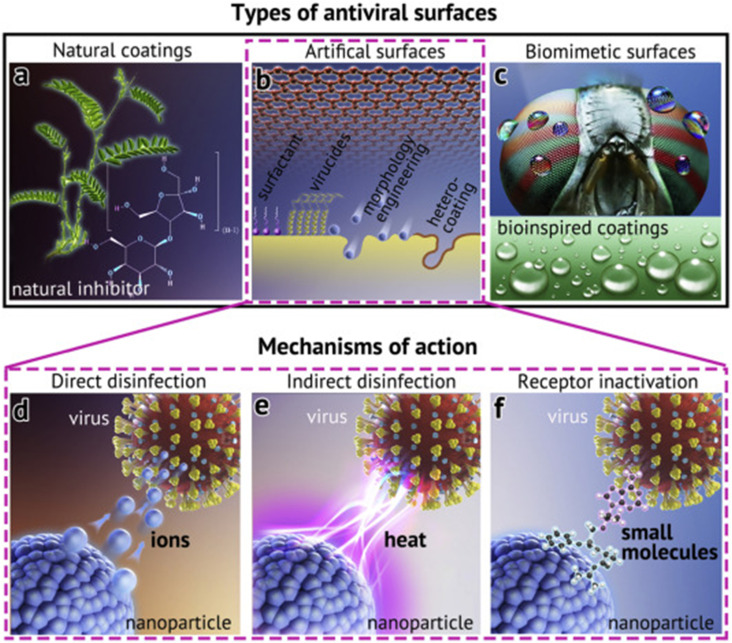
(a)–(c) Different types of antiviral coatings and [(d)–(f)] their mechanisms of action: Reprinted with permission from Z. Sun and K. Ostrikov, Sustainable Mater. Technol. **25**, e00203 (2020). Copyright 2020 Elsevier.

Primarily, the exposed surfaces are contaminated due to the viral adhesion/colonization and subsequent proliferation with the formation of biofilms.^8^ The effect of surface contamination can be severe in the case of SARS-CoV-2. It is advocated that the coronavirus can survive on a variety of surfaces for tens of hours to seven days,^9^ although such surface contamination can be removed by utilizing the traditional disinfecting cleaning method, which, unfortunately, is just a temporary relief.^10^ The bioburden level on the cleaned surface returns to the state of precleaned surface within 2.5 h.^10^ Therefore, it is envisaged to develop an active surface with an ability to combat viral adhesion/colonization and its further proliferation. Sun *et al.* have provided an extensive review on antiviral surface coatings and their mechanism of action. The authors have classified the anti-infective surface as natural coatings, artificial surface, and biomimetic surfaces with the mechanism of action as direct disinfection, indirect disinfection, and receptor inactivation, as illustrated in Fig. 2.^11^ In addition, the team of experts led by Weiss *et al.* reviewed different nanotechnology enabled approaches against the COVID-19 pandemic.^12^ In another detailed review, Singh *et al.* have identified such anti-infective surfaces as contact killing and antimicrobial agent eluting.^13^ The authors have exploited a number of pathways to attain effective multifunctional or monofunctional surfaces that include the adhesive mediated approach, coatings with anti-infective metals, photosensitized coatings, or enzymatic activated coatings.^13^

Among these approaches, use of nanoparticles such as silver, gold, copper, zinc oxide, titanium dioxide, and carbon-based nanotube and bionanoparticles such as chitosan is confirmed to be immensely effective for antiviral applications due to their increased contact with microbes by virtue of their small sizes (1–10 nm, especially for nanoparticles).^14^ It is important to note that the field of surface coating is already fairly advanced with commercially available coating materials in the form of “smart coatings,” “multifunctional coatings,” or even “monofunctional coatings” for a number of applications. However, technologies related to the antimicrobial coatings are still in their infancy and not yet commercially realized. There are several considerations that have hindered the commercialization of anti-infective surfaces or coatings: (i) nanoparticle cytotoxicity and biocompatibility to human cells are still debatable, (ii) lack of international standardization, (iii) lack of mechanical robustness and its effectiveness for a wide range of microbes, and (iv) ambiguity in commercial and economic viability for mass market application.^13^

### A. Metal-based coatings

**FIG. 3.**
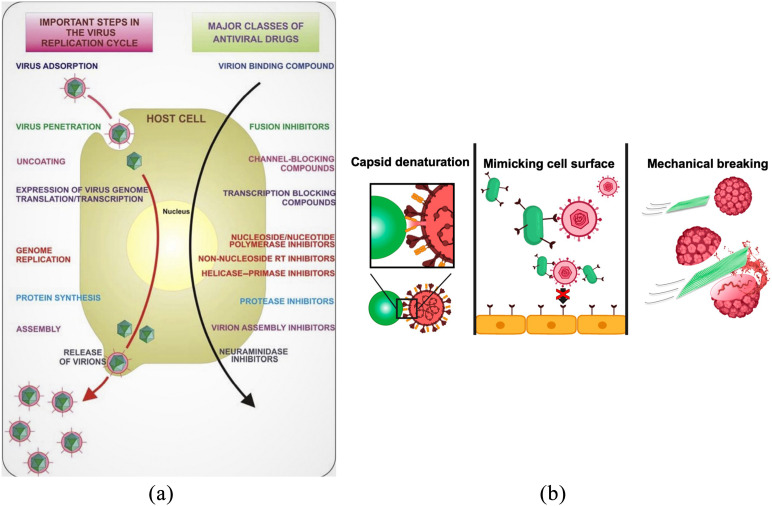
(a) Key steps in the virus replication cycle that provide antiviral targets.^15^ (b) Mechanism of blocking virus entry into host cells.^16^

Human interaction with the virus undergoes a particular sequence and is broadly similar for most of the viruses. Galdiero *et al.* have demonstrated that each sequence of virus replication facilitates an opportunity for their inhibition, as shown in Fig. 3(a).^15^ It is known that targeting viruses at the early stages can be a promising approach due to its ability to inhibit them extracellularly. In this context, it is believed that a smart surface developed from metallic nanoparticles can be a paramount inhibitor to curtail formation of viral colonization and its further spread. Reina *et al.* have reported that interaction of the virus with metallic nanoparticles can facilitate early blocking of viral entry to the host cell by virtue of blocking the targeted protein for viral entry, capsid protein oxidation, cell surface mimicking, or mechanical rupture of viruses, as shown in Fig. 3(b).^16^

The effectiveness of nanoparticles can be realized by the mechanism such as the production of reactive oxygen species (ROS), cell wall/membranes disruption, interruption of energy transduction and enzyme activity inhibition, and DNA damage.^17^ Although there are number of nanoparticles utilized for therapeutic applications, silver, gold, and copper are the most well-known antimicrobial agents.

#### 1. Silver nanocoating

Among various nanoparticles, silver nanoparticles have demonstrated substantial efficacy against bacteria, viruses, and even eukaryotic organisms.^18^ Therefore, silver nanoparticles are exploited against a number of viruses such as human immunodeficiency virus (HIV), respiratory syncytial virus, and hepatitis B virus, either in the pristine particle form or encapsulated with mercaptoethane sulfonate (MES), poly *N*-vinyl-2-pyrrolidone (PVP), polysaccharide, etc.^19^ For instance, Kumar *et al.* have developed environmentally friendly silver nanoparticle embedded paint from common household paint.^20^ It is believed that available silver ions and metallic silver in coating synergistically contributed to its antimicrobial activity. Silver nanoparticle coated polyurethane condoms have also been developed to inhibit infectious viruses such as HIV and Herpes simplex virus (HSV).^21^ The silver nanoparticle coatings were found to be substantially stable and they do not disrupt the primary nature of the polyurethane surface. It was claimed that the coated surface provides an additional line of defense against sexually transmitted diseases and possesses the ability to directly damage the viruses. The work of Sreekanth *et al.* has demonstrated excellent antiviral efficacy of silver nanoparticles against influenza A virus. In this study, silver nanoparticles were synthesized through the aqueous extract from Panax ginseng via the green ultrasonication route.^22^ Engineered silver nanoparticles are also a potential pathway for enhancing their antiviral activity. In this context, studies have been reported to modify silver nanoparticles with other moieties such as materials,^23^ oseltamivir,^24^ graphene oxide (GO),^25^ zanamivir,^26^ and aminoadamantane^27^ to inhibit viruses and activate the innate immune response system.^25^ Essentially, the antiviral efficacy of silver nanoparticles is broadly evaluated in liquid environment, and few studies have been reported to develop antiviral surface coatings.^28^ In addition, the antiviral activity of the silver nanoparticles is found to be dependent on their size, shape, stability, and capping agents.^29,30^ As a general observation, Lara *et al.* have revealed that the therapeutic index of the silver ion for HIV-1 available in silver salts is 12 times lesser than the silver nanoparticle.^31^ The tendency of self-agglomeration of silver nanoparticles and related environmental pollution is still a considerable concern, which drastically diminishes its antiviral efficacy and needs to be investigated further.^16,32^

#### 2. Gold nanocoating

The therapeutic value of gold nanoparticles has been known for 2000 years. Gold is still the preferred material in medical applications, compared to its contemporary silver due to its low toxicity for healthy cells.^33^ The ameliorated medicinal value of gold nanoparticles can be realized from the work of Quach *et al.* where authors have developed a hybrid subunit vaccine [gold nanoparticles and domain III of the envelop protein (EDIII)] against dengue viruses.^34^ Gold nanoparticle-based vaccines have also been studied as an alternative solution to acute respiratory syndromes such as that caused by coronavirus.^35^ Halder *et al.* have evaluated the synthesis of quasi-spherical mono dispersed gold nanoparticles for inhibition of Herpes simplex virus (HSV) infections.^36^ The authors have elucidated that gold nanospheroids have exhibited excellent antiviral efficacy where viral inhibition was attained by invasion of gold nanoparticles into infected Vero cells. Facile surface modification of gold nanoparticles was proceeded by their conjugation with drugs and ligands. Bowman *et al.* have reported that conjugating gold nanoparticles with mercaptobenzoic acid constructs a multivalent therapeutic against the fusion of HIV-1 with human T-cells. The authors have advocated that conjugating gold nanoparticles possess the ability to transform inactive weakly binding monovalent molecules into highly active drugs.^37^ Conjugating gold with oligonucleotides have been also revealed that such conjugations are not toxic to healthy cells.^38^ Apparently, the antiviral activity of gold nanoparticle is accepted to be associated with the inhibition of the hemagglutinin (HA) glycoprotein. The review of Skehel and Wiley provides detailed insight into influenza hemagglutinin and stimulated them as a target for neutralizing antibodies.^39^ The proposed strategy was further exploited by Kim *et al.* where porous gold nanoparticles were used to curtail the influenza A virus.^40^ It was confirmed that high affinity of porous gold nanoparticles toward the disulfide bond facilitates the bond cleavage and subsequent disruption in fusion of the virus in host cells.

#### 3. Copper nanocoating

Copper based materials have a long history in biocidal applications. The study of Warnes *et al.* has asserted that human coronavirus 229E (HuCoV-229E) on a copper surface is inactivated in less than 30 min if the copper percentage in the surface alloy is more than 90%.^41^ The authors have postulated the generation of superoxide and hydroxyl radicals as the paramount inhibition mechanism, but the phenomenon of direct killing is also activated when the surface is developed from 100% copper.^41^ It is believed that SARS-CoV-2 viruses can only last up to 4 h on the copper surface, while viruses were detected up to 72 h on stainless steel surfaces.^42^ Apart from metal nanoparticles, solid-state inorganic materials such as metal oxides have also been observed to be effective due to their ease of use and chemical robustness.^43^ Antiviral effectiveness of the ionic forms of solid state copper including CuO, Cu_2_S, CuCl, and CuI has been reported in the case of bacteriophages and bacteria,^44^ whereas those of solid-state cupric compounds are markedly lower.^45^ On a Cu_2_O-loaded glass substrate, for example, the infectious activity of bacteriophages and bacteria was reduced by five orders and three orders, respectively, but no significant reduction has been traced in CuO-loaded substrates. It is confirmed that Cu_2_O denatured and adsorbed more proteins than CuO, and infectious deactivation is performed following direct contact with the solid-state surface of cuprous compounds, but not reactive oxygen species or copper ions.^44^

#### 4. Titanium-based coatings

Titanium based structures are one of the most popular photocatalysts used due to their great photo-oxidation of organic compounds, excellent chemical stability, strong oxidizing power under UV radiation, and excellent chemical resistance and photostability. For efficient decomposition purposes, including deodorizing and antibacteriality in living and working environments, only the presence of light particularly in UV range is required. It is also confirmed that TiO_2_ has the potential to destroy both gram-positive and gram-negative bacteria, including various viral species and parasites.^46^

**FIG. 4.**
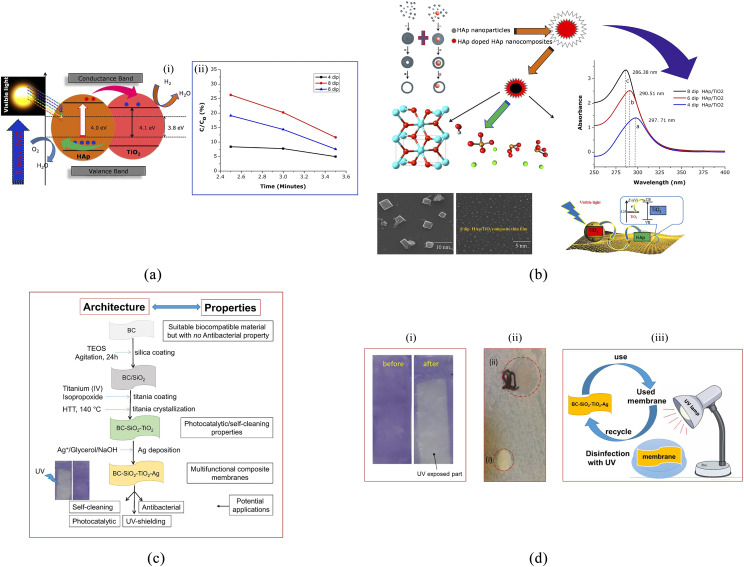
(a) Photocatalytic activities of HAp/TiO_2_ composite thin films (i) and the plot of time vs C/C_o_. (b) Schematic diagram of photocatalytic activities of HAp/TiO_2_ composite thin films: a—4 dip, b—6 dip, and c—8 dip.^54^ (c) Schematic representation of the SiO_2_–TiO_2_ membranes surface. (d) Confirmation of the self-cleaning activity of these membranes under UV illumination for 50 min (i), (ii) antibacterial activity of the BC–SiO_2_–TiO_2_ (red dotted circle at the bottom) and SiO_2_–TiO_2_/Ag nanocomposites against Kluyvera (gram-negative, red dotted circle at the top), and (iii) schematic representation of the UV-induced disinfection of the used membrane.^67^

The photocatalytic activity of TiO_2_ has demonstrated limitation in large scale applications due to wide bandgap and high electron–hole recombination rate. Doping of TiO_2_ with transition metal ions or anions has been performed by several research groups around the world to synthesize highly efficient visible-light-sensitive photocatalysts. These efforts have been challenged due to their low quantum efficiencies (QEs) caused by the carrier recombination centers in metal-ion-doped TiO_2_ or the low oxidation power and mobility of photogenerated holes in non-metal-doped TiO_2_.^47,48^ In many cases, a doping system has a direct effect on shifting the photoresponse to the visible range.^48–51^ It is also reported that noble metal doping (Pt^IV^, Ir^IV^, Rh^III^, Au^III^, Pd^II^, Co^II^, and Ni^II^) on TiO_2_ extends the light absorption into the visible range^52^ but caused a decrease in photocatalytic performance dramatically.^48^ In a study by Liu *et al.*,^53^ the surface doping of TiO_2_ with Cu(ii) or Fe(iii) nanoclusters increased the visible-light sensitivity of the resulting material without inducing impurity levels in the bandgap. Exploring the simple and versatile methods such as sol–gel for thin film preparation attracted significant interest due to its advantages such as low processing temperature, homogeneity, the potential large area coating, and cost-effectiveness compared to other techniques. Studies on sol–gel formation of hydroxyapatite/titanium dioxide composite thin films of different dipping cycles have revealed good inhibition on gram-positive and gram-negative bacteria^54^ [see Figs. 4(a) and 4(b)].

Other strategies have been explored to solve the limitation of TiO_2_ for photocatalytic application. This includes coupling with narrow bandgap semiconductors or carbonaceous materials as sensitizers.^55^ Carbonaceous materials with a specific sp^2^/Sp^3^ graphitic structure have been widely studied in many fields, such as catalyst supports, fillers, adsorbents, and battery electrode material,^56–66^ and demonstrated its potential to reduce the TiO_2_ bandgap and shift within the visible range while maintaining the photocatalytic performance. This presents a great opportunity to use carbon synthesized from sustainable resources that have direct impact on the cost effectiveness of the system. Carbon nanotubes, graphene, graphene oxide, and carbon quantum dots have been used extensively for the development of TiO_2_–carbon photocatalysts. It is believed that carbon can accelerate the charge transfer from the TiO_2_ structure to the surface area of oxidation reaction while enhancing the conductivity.^55^ It is believed that the hybrid structures of TiO_2_ and other carbon materials with tunable surface area are highly promising for the development of high performance photocatalysts for everyday life by controlling the carbon resources to low cost precursors and establishing the versatile and easy-to-apply methods for the fabrication of photocatalysts.

As a case study, organic–inorganic hybrid membranes (BC–SiO_2_–TiO_2_/Ag) based on bacterial cellulose (BC) that contain photoactive (TiO_2_) and antibacterial (Ag) components have been developed through coating of BC with silica and crystalline TiO_2_.^67^ The prepared photoactive BC–SiO_2_–TiO_2_ membranes exhibited excellent TiO_2_-loading dependent photocatalytic/self-cleaning activity toward crystal violet dye deposited as an overlayer on the surface of the membranes, degrading 97% of the dye within 50 min of UV illumination [see Figs. 4(c) and 4(d)].

### B. Carbon-based coatings

**FIG. 5.**
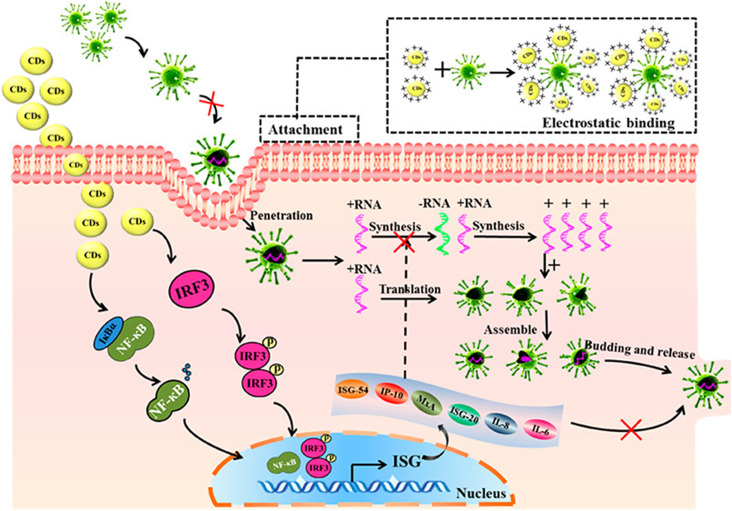
Antiviral activity of curcumin derived carbon dot nanoparticles: Reprinted with permission from Ting *et al.*, ACS Appl. Nano Mater. **1**(10), 5451–5459 (2018). Copyright 2018 American Chemical Society.

Carbon-based nanostructures are another class of antiviral agents, which are extensively exploited for biomedical applications due to their excellent physiochemical and medical diagnostic characteristics.^68^ The unique ability of carbon atoms to form different allotropes makes them an ideal material for biomedical applications. Carbon can also present different dimensionalities such as 0D Buckyball or carbon dots, 1D carbon nanotube, and 2D graphene or graphene oxides and can be further stacked into 3D graphitic sheets.^59,60,69,70^ Among various carbon nanomaterials, carbon dots (CDs) have manifested promising antiviral attributes. CDs have been found to be environmentally benign with no toxicity to *in vitro* and *in vivo.* Additionally, the photoactivated antiviral characteristics of CDs have enticed a number of research activities.^71^ Carbon dots are primarily less active against viruses *in vivo*, but functionalization offers further opportunities for augmenting antiviral efficacy. For example, Ting *et al.* have synthesized stable cationic carbon dots from curcumin (Ref. 72). They synthesized nanoparticles that suppress viruses by the synthesis of negative-strand RNA in addition to the formation of interferon-stimulating genes (ISGs) and proinflammatory cytokines, as illustrated in Fig. 5. The elaborated antiviral mechanism of CDs against viruses has been also supported by the studies of Du *et al.*^73^ There are number of other functionalization moieties such as 2,2′-(ethylenedioxy) bis(ethylamine) (EDA), 3-ethoxypropylamine (EPA),^74^ boronic acid,^75^ and amino phenylboronic acid,^76^ which are utilized for surface modification of CDs.

Another nanocarbon family, Fullerene, was first reported as an antiviral agent in 1993 by Friedman *et al.*^77^ and Sijbesma *et al.*^78^ However, the antiviral adequacy of fullerene is not fully exploited due to its nature of hydrophobicity and insolubility in water.^79^ Therefore, efforts have been made to render water soluble fullerene moieties.^79^ Goodarzi *et al.* have briefly reviewed the inhibitory activity of fullerene and its derivatives against HIV.^80^ It is apprehended from their work that virostatic or virucidal tendency of fullerene and its derivatives are associated with the site of functionalization, position of the side chain, and type of derivatives. A comparison of various nanoparticles including nanocarbons is provided in Table I.

**TABLE I.** Inhibition mechanism of different nanomaterials against viruses.

Nanomaterials	Type of viruses	Inhibition mechanism
Silver nanostructure	HIV-1, HSV-1, Influenza virus,Tacaribe virus, SARS-CoV-2	Interaction with hemagglutinin
		Preventing viral attachment
		Cell binding/penetration
		Inhibiting accumulation of
		reactive oxygen species
Gold nanostructure	HSV, HIV-1, Influenza A	Inhibition of hemagglutinin
		Electrostatic attraction of negatively
		charged bilayer of cell membrane
		Production of reactive oxygen species
Copper nanostructure	Influenza, Hepatitis A,	Generation of reactive oxygen species
	Feline calicivirus, adeno virus	
Titanium nanostructure	H1N1 Influenza A, adeno virus,	Generation of reactive oxygen
	HSV-1, Influenza virus,	species (ROSs)
Carbon-based nanoparticle	Ebola, Dengue, Zika, Human coronavirus 229E, African swine flu	Cell wall penetration
		Agglomerate formation
		Reactive oxygen generation
		Interaction of negatively charged
		surface to positively charged capsid
		Mechanical disruption of capsid

## III. ANTIVIRAL COATINGS AGAINST SARS-CoV-2

Given the growing number of infected patients and the possibility of evolution of the present SARS-CoV-2 to a stronger version have forced the scientists and decision makers to adopt new strategies that concentrate mostly on novel designs with antimicrobial and antiviral properties.^81^ The SARS-CoV-2 virus is highly stable, viable, and potentially infectious on various types of surfaces, including metals, woods, glasses, and plastic and fabric surfaces. It is known that the SARS-CoV-2 virus can stay stable for several days. However, it can be simply destroyed by breaking the delicate envelope around the virus using disinfectants such as ethanol (62%–71%), hydrogen peroxide (0.5%), or sodium hypochlorite (0.1%).^82^ The highly desirable alternative would be antiviral surfaces that repel the pathogens (thus, the virus faces a non-sticking surface)^83^ or the development of antiviral surfaces that can sanitize itself by rapid neutralization of pathogens.^84^ One of the strategies to develop antimicrobial surfaces is through surface coating by polymer composites containing nanoparticles, which intrinsically have antiviral and antimicrobial properties similar to silver nanoparticles.^30,85^ Regarding the protection mechanism of coating, the virus either can be effectively blocked or destroyed within a certain time.

### A. Hybrid coating materials against SARS-CoV-2

The synthesis of antiviral and antimicrobial coating materials provides the opportunity for the development of high quality and effective air fileting systems. Since non-woven textiles are widely used in face masks to prevent airborne transmission, possessing a layer of the antiviral/antimicrobial coating in facemasks provides an effective protection layer to suppress the transfer of the aggressive viruses to the respiratory system. In a recent work that focuses on the SARS-CoV-2 pandemic, a hybrid of silver nanocluster/silica coating was deposited onto the surface of disposable face masks using the sputter coating technique.^86^ The well-embedded silver nanoparticles in the silica glass substrate facilitated the conformal deposition of the silver nanocluster/silica composite onto the fibers.^86^ The deposition process was accompanied by the changes in the surface color of disposable masks. The tests toward the evaluation of effectiveness of a coated face mask confirmed the absorbance of inoculum via coated mask, while the inoculum remained on the surface of non-coated mask for a long time. In addition, it was confirmed that the inoculums were dried on the surface of silver nanocluster/silica hybrid mask, while they remained untouched and active on the surface of a non-coated mask.^86^ Virus infectivity tests on coated and uncoated face masks showed that the uncoated mask has the highest infectivity, while the level of infectivity was reduced (by one order of magnitude) on the surface of a composite film with 3 wt. % silver nanoclusters.^86^

Metal nanoparticles and ionic species have been found as potential materials to combat COVID viruses.^87–90^ The mechanism of interaction of nanoparticles with viruses can be divided into two main groups. In the indirect interaction, nanoparticles do not directly impact the viruses; instead, they will intensify the antiviral activity of agents. In the indirect mechanism, nanoparticles are employed to transport, increase the stability, and enhance the bioavailability of antiviral agents.^91^ In the direct activity mechanism, the nanomaterials deactivate the virus by altering their viral structure or by changing the genetic structure of materials.^91^ As an example, the silver nanoparticles may attach to the surface of glycoproteins on the virus and then reduce the fusion process by reducing its ability to attach to the cells.^92^ The other mechanisms are also proposed for the reaction between nanoparticles and viruses. It was observed that the ultra-structure of virus is affected by the iron oxide and nanoparticles where the broken viral particle causes the vertical inhibition of enveloped and naked particles.^93^

A study conducted by Fujimori has shown that the surfaces coated with metal ions and nanoparticles have a considerable impact on the infectivity of lentiviruses (from HIV family). It was confirmed that the surface coated with copper (I) iodide nanoparticles strongly blocked the cell infection caused by the viruses, especially SARS-CoV-2.^89^ This promising finding has an opportunity for the development of new antiviral coatings based on polymer materials containing ionic copper and other metal nanoparticles, which can be easily sprayed on as paint or plastic film covers onto various surfaces.^30,88^ Through precise control of the amount of nanoparticles present in coating materials and adjustment of the abrasive characteristics of coating, durability and antiviral effectiveness of protective layers can be vastly improved. One of the main challenges in the production of antiviral polymeric composite materials with metal particles lies in the tendency of metallic nanopowders to oxidize.^94^ Nevertheless, considering the high surface area to volume ratio of nanoparticles, a relatively small amount of nanoparticles facilitate a high level of antiviral characteristics. Furthermore, since nanoparticles are already embedded in the polymeric matrix, they can be preserved from oxidation and the composite film can keep its antiviral properties for a longer period.

It has been demonstrated that the metal and metal oxide nanoparticles similar to zinc oxide nanoparticles,^85^ cuprous oxide nanoparticles,^87^ silver nanoparticles,^30,88^ nanosized copper (I) iodide particles,^89^ gold nanoparticles on silica nanoparticles (Au–SiO_2_ nanoparticles), and quaternary ammonium cations (QUATs)^90^ are highly promising materials to inactivate viruses. In a similar concept, an antibacterial coating has been developed based on non-migratory QUATs and positively charged silver nanoparticles as bioactive nanoparticles, which are dispersed in the polymer matrix.^95^ The synthesized antimicrobial coating has an extremely low surface energy value (>20 mN/m), and thus, it behaves as an omniphobic surface and repels water and oily components from the surface.^95^ The measured contact angle on the surface of coated surfaces were found to be >130° and >50° for water and hexadecane products, respectively. The developed coating can effectively repel (up to 99%) and inactivate the family of coronavirus on the surfaces and mitigate its spread via direct human contact of surfaces.^15^ It is believed that silver nanoparticles can inhibit the replication of virus nucleotides. In this mechanism, the electron donor groups are bound to metallic nanoparticles and enzymes, effectively incapacitating the energy source of the cell and thus leading to the death of microbes.^95^ In addition, it is confirmed that the cationic silver nanoparticles and QUATs inactivate the SARS and COVID-2 by interaction with the surface spike protein (S protein). The results of evaluation of developed antiviral compounds confirmed an antiviral efficacy of 99.9% in just 2 h of contact with the surface. Moreover, an antiviral test is currently in progress to establish its efficacy on the inactivation of SARS-CoV-2 on different surfaces to stop the secondary spread from various surfaces to living cells through touch.^95^

### B. Atomic layer deposition for antiviral surfaces

**FIG. 6.**
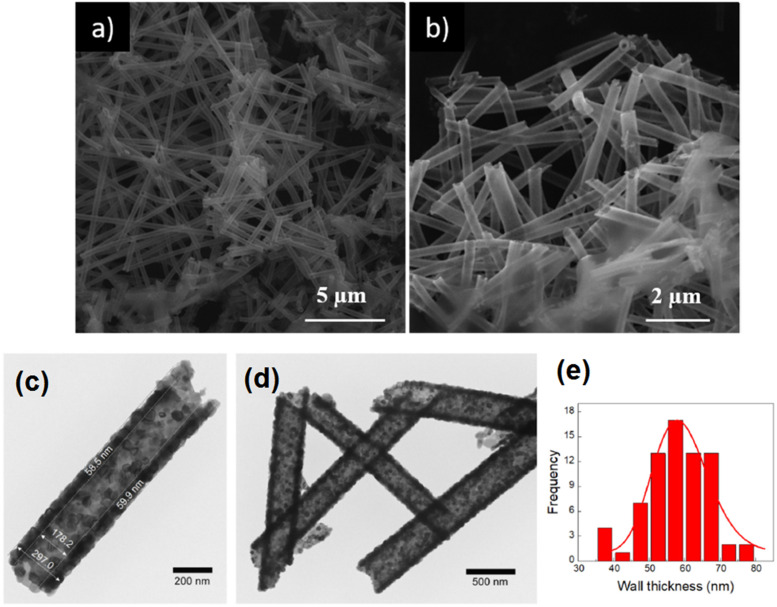
(a) The SEM micrograph of ZnONT. (b) The TEM images of ZnONT (500 ALD cycles, calculated at 450 °C) at different magnifications. [(c)–(e)] ZnONT wall thickness histogram. Reprinted with permission from López de Dicastillo *et al.*, Nanomaterials **10**(3), 503 (2020). Copyright 2020 MDPI.

Generally, atomic layer deposition (ALD) has outstanding potential for the deposition of metal oxide films and catalytic substrates with antiviral properties.^96^ As an example, ZnO nanoparticles have demonstrated effective antimicrobial properties that arise from their effects on improved cellular internalization of bacteria and viruses.^97^ Considering the geometrical features of nanostructured oxides, the hollow ZnO nanotubes and nanorods are categorized as the highly efficient metal oxide nanostructures with antiviral and antimicrobial properties.^98–100^ Atomic layer deposition (ALD) as a cyclic vapor-phase deposition process takes the advantages of temporarily separated and self-limiting reactions of two or more reactive precursors^101^ and allows the deposition of nanometer-thick layers of materials on substrates.^102^ The sequential interactions of chemical precursors that are usually metal–organic precursors and a co-reactant as reducing or oxidant agents on the substrate surface allow the formation of ultra-thin atomic scale monolayers of thin films.^30^ The most important advantage of ALD is the capability of the technique for conformal and flawless ultra-thin films over complicated three dimensional (3D) structures. In this technique, the surfaces of the most complicated structure and geometry can be used for deposition as long as the chemical molecules can diffuse into the surface. Considering the size of chemical molecules and reactants, the technique is capable of deposition of antiviral materials onto interwoven fibrous structures, which makes it a suitable method for antiviral coating on respiratory masks. In a recent report, the ALD technique was used for the deposition of zinc oxide nanotubes onto electrospun polyvinyl alcohol nanofibers, followed by polymer removal through calcination, which led to an antimicrobial nanostructure with a high surface area.^103^ The microstructural studies have confirmed the development of uniformly distributed ZnO nanotubes after thermal annealing (Fig. 6). It was found that thermal annealing at 450 °C for 45 min is a practical approach for the removal of PVA nanofibers, which led to highly homogeneous hollow ZnO nanotubes.^103^ To measure the antibacterial properties of the synthesized material, a bilayer nanocomposite composed of ZnO nanotubes–Acry/PE bilayer films was fabricated and tested against bacteria sources. The results have confirmed the improved antibacterial activity of ZnO nanotubes compared to that of Zn nanoparticles. Both antimicrobial coatings with 1 wt. % ZnO nanotubes and ZnO nanoparticles demonstrated a great antibacterial activity with the capability of inhabitation of bacteria. Compared with the bilayer system containing commercial zinc oxide nanoparticles, materials with zinc oxide nanotubes presented higher antimicrobial effectiveness, since their tubular morphology presented a higher specific surface area and lower aggregation than commercial spherical zinc oxide nanoparticles.

### C. Spray coating

One of the main strategies for longer protection of surfaces from the infection of SARS-CoV-2 is the development of long lasting antiviral coating materials that can be easily sprayed onto the surfaces. The spray coating technique is a versatile and inexpensive approach that is the most common strategy used by the governments for disinfection and protection of public surfaces,^104^ for example, antimicrobial coated surfaces with SurfaceWise2™ (a quaternary ammonium polymer coating) prepared and tested against human coronavirus.^105^ The results of the developed antiviral surface coating showed the effectiveness of the spray coating technique to reduce the concentration of the viruses by greater than 90% in 10 min and greater than 99.9% after 2 h of contact. The coating formulation when tested in suspension yielded a greater than 99.99% reduction of HCoV 229E within 10 min of contact. This outcome presents an opportunity to control the transmission of SARS-CoV-2 from contaminated fomites.^105^ In another example, a polymer based multilevel antimicrobial (MAP-1) coating was developed by the Hong Kong University of Science and Technology (HKUST), which is highly capable of inactivating and kill bacteria and viruses, including SARS-CoV-2. MAP-1 coating acts as an effective protective layer against microorganisms where it was found that 98.7% of viruses and bacteria were eliminated after three weeks in a hospital environment. The service life of MAP-1 was confirmed after a long-term usage of the polymer coatings for 90 days.^106^ In a recent work in Poland, the Titan Solid product made by Lumichem Company developed a TiO_2_ based antiviral coating. It is claimed that the developed material effectively eliminates pathogenic microorganisms—bacteria, fungi, viruses, and their spores, building a shield that remains active for a minimum of one year from the first application.^107^

Some other coating strategies focus on the development of super-hydrophobic nanocoatings to combat the transmission and the spread of the viruses, including encapsulation, contamination, suppression, and elimination.^108^ In developed superhydrophobic nanocoatings, the elimination of the COVID virus will be through the use of antiviral and antibacterial copper nanoparticles or dedicated copper surfaces.^76^ A flexible superhydrophobic surface can be fabricated by dispersing hydrophobic nanoparticles such as silica in a flexible polymeric matrix, such as silicone.^109^ In this work, the successful dispersion of hydrophobic nanoparticles can be assured by the aid of a solvent (e.g., acetone). The produced emulsion can be spray-coated onto the desired surfaces and textile to create a superhydrophobic surface.^110^ A nanocoating was developed based on 30 wt. % silica nanoparticles in the matrix of siloxane-modified epoxy.^110^ The superhydrophobicity of the surface was well examined and confirmed. The water droplet was immediately repelled from the surface of a superhydrophobic textile covered with a polymeric nanocomposite. This nanocoating was found highly effective for the development of superhydrophobic coatings on metal, glass, wood, and fabric substrates. The presence of nano/micro-asperities superposed on the main surface asperities is one of the main characteristic features of superhydrophobicity.^110^

**FIG. 7.**
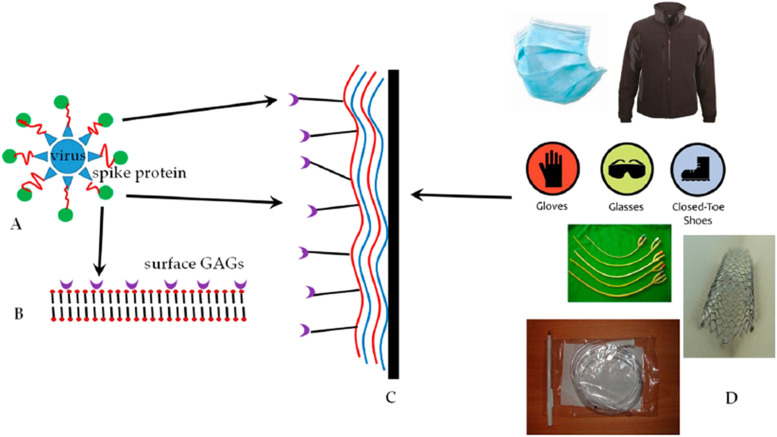
(a)–(d) The capture of the coronavirus family on the surface of GAGs.^108^

In another technique, a substrate that undergoes layer-by-layer (LbL) nanocoating was fabricated with antiviral properties.^108^ In this approach, glycosaminoglycans (GAGs) as polysaccharides were employed to develop the layer by layer nanocoating. It was confirmed that the spike proteins of the coronavirus are capable of binding to GAGs on the ACE-2 receptor of the lung parenchyma (Fig. 7) and that the coronaviruses can be nanocaptured by GAGs. The same strategy can be employed to cover the surface of textile and medical devices to capture the coronavirus.

### D. The potential of graphene against SARS-CoV-2

Graphene is from the family of 2D materials with extraordinary physiochemical and mechanical characteristics, which are beyond the limitation of three-dimensional graphite. Graphene research for the development of biocompatible materials, drug delivery application, and drug resistance detection is currently enjoying the spotlight. Graphene has also presented antimicrobial behavior, including trapping or deactivating of the bacteria due to its very high surface area.^111,112^ One of the first antiviral activities of the graphene-based structure was observed in the interaction of thin films of graphene oxide ribbons (rGOs) with tungsten oxide via photo-activation of bacteria phases under visible lights.^113^ It is believed that the extraordinary large surface area of 2D graphene provides a unique platform for the highest number of ligand contacts for the adsorption of negatively charged sulfates and then facilitates the interaction with positively charged residues to block the microorganisms.^114^ It has been shown that the graphene oxide (GO) flakes can successfully wrap and confine microorganisms by enclosing them in an insulating carbon blanket.^115^ The mechanism of interaction of graphene in contact with virus is based on hydrogen bonding, electrostatic interactions, and redox reaction.^116^ The graphene derivatives have been investigated for drug delivery in antiviral compounds similar to reverse transcriptase inhibitors conjugated with graphene quantum dots to treat HIV^117^ and hypericin–GO against reovirus.^118^ Graphene is also capable of successfully capturing particulates and bacteria, which substantially decreases the spread and transmission of infections.^119^ It has been shown that the graphene-based filters are able to efficiently block the bacteria. These filters are capable of following heat treatment, and thus, they can be tempered at higher temperatures to destroy the bacteria and disease agents.^120^ GO films have also been used as the breathable barrier in fabrics.^121^ The hydrophobic characteristics of the fabrics can also be achieved by graphene-based coating of fabrics. The hybrid of graphene oxide and other nanoparticles such as silver has demonstrated antiviral performance.^25^ The specific properties of graphene can be accompanied by the antibacterial effects of other silver and titanium oxide nanoparticles to make graphene composites containing nanoparticles with antiviral characteristics to trap and eradicate the SARS-CoV-2 families.^121,122^

A silver nanoparticle–graphene oxide (GO-AgNPs) nanocomposite was synthesized via interfacial electrostatic force^25^ to be used as an antiviral structure. The results indicated that exposure with GO-AgNPs nanocomposites could obviously suppress porcine reproductive and respiratory syndrome virus (PRRSV) infection that is more effective when compared to sole AgNPs and GO. It is confirmed that the GO-AgNPs antiviral agent improves the production of interferon-α (IFN-α) and IFN-stimulating genes (ISGs), favorable to inhibiting the proliferation of virus.^25^

In another work, a superhydrophobic, photo-sterilize, and reusable mask based on a graphene nanosheet-embedded carbon (GNEC) film exhibits high hydrophobicity (water contact angle: 157.9°) and filtration efficiency [with 100% bacterial filtration efficiency (BFE)].^123^ In addition, the GNEC mask presents a photo-sterilize ability to being heated up to 110 °C quickly under the solar illumination, which provides promising potential for further investigation.

**FIG. 8.**
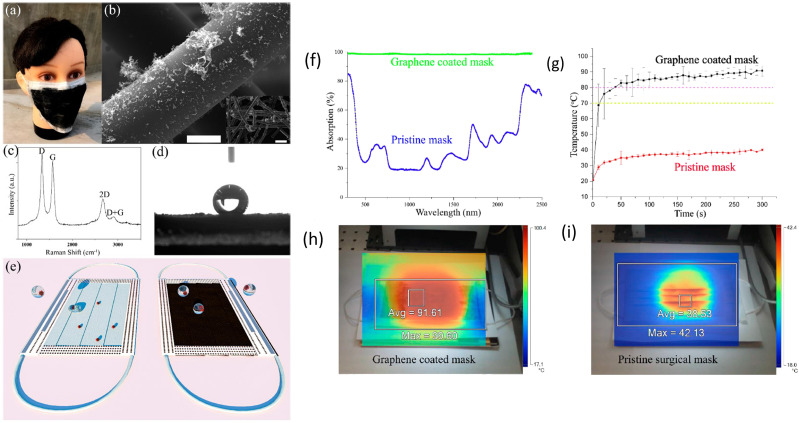
(a) Laser-fabricated graphene mask. (b) SEM of the graphene-coated nonwoven fiber within the surgical mask [(c) and (d)] Raman spectrum and water contact angle of the graphene-coated mask. (e) Illustration of the self-cleaning properties compared to the pristine blue mask (left). Photothermal performance of the masks. (f) Optical absorption. (g) Surface temperature measured by using infrared camera (h) and (i) after 5 min of solar illumination. Reprinted with permission from Zhong *et al.*, ACS Nano **14**(5), 6213–6221 (2020). Copyright 2020 American Chemical Society.^124^

Recently, graphene has been used to develop self-cleaning masks with the use of the dual-mode laser-induced forward transfer method for depositing few-layer graphene onto the nonwoven masks.^124^ Superhydrophobic characteristics were confirmed on graphene coated mask surfaces, which makes the incoming aqueous droplets to bounce off from the surface of mask. The surface temperature of the mask can quickly reach to over 80 °C under sunlight, making the masks reusable due to sunlight sterilization^124^ (Fig. 8). While the ordinary face masks have low absorption toward sunlight, the graphene-coated masks show over 95% absorption across the whole solar spectrum from 300 to 2500 nm. As SARS-CoV-2 is sensitive to heat, development of photothermal graphene-coated masks with a promising self-sterilization feature provides great hope for large scale fabrication of personal protection equipment (PPE) effective for the fight against coronavirus.

## IV. SUMMARY AND OUTLOOK

In response to the SARS-CoV-2 global health outbreak, we have summarized the current state of knowledge in antiviral coating materials as well as possible nanocoating to prevent the transmission of the transferrable SARS-CoV-2. The exposed surfaces are contaminated due to the viral adhesion/colonization and subsequent proliferation with the formation of biofilms. Surface contamination is currently eliminated by utilizing the traditional disinfecting cleaning method, but studies reveal that disinfecting provides temporary relief. Promising works have been performed in the field of antiviral coating and further research is undoubtedly required. It is believed that nanomaterials including metal oxide nanostructures, graphene, CNTs, carbon quantum dots, and titanium dioxide and bio-nanoparticles such as chitosan, capped silver, graphene, gold, and silicon nanoparticles could play a leading role in the development of antiviral coatings. The ease of use, low toxicity, health issues, long lasting efficiency, and sustainable fabrication are some of the main factors that need to be considered when developing the potential coating materials. The world is going through a challenging time and providing a single solution for all types of surfaces is cumbersome, but it is possible to develop novel solutions for surface coating based on currently available research studies and commercial products. Rapid development of antiviral surface coating materials would certainly benefit from the multidisciplinary collaboration between materials science, chemistry, and environmental and biomedical sciences.

## Data Availability

Data sharing is not applicable to this article as no new data were created or analyzed in this study.

## References

[1] Y.-C. Liu, R.-L. Kuo, and S.-R. Shih, “COVID-19: The first documented coronavirus pandemic in history,” Biomed. J. 43, 328 (2020). 10.1016/j.bj.2020.04.007 32387617 PMC7199674

[2] See https://www.euro.who.int/en/health-topics/communicable-diseases/influenza/pandemic-influenza/past-pandemics for World Health Organisation.

[3] T. D. Brock *et al.*, “Viruses and virology,” in Brock Biology of Microorganisms (Prentice-Hall, Upper Saddle River, NJ, 2003), Chap. 9.

[4] E. A. Almand, M. D. Moore, and L.-A. Jaykus, “Virus-bacteria interactions: An emerging topic in human infection,” Viruses 9(3), 58 (2017). 10.3390/v9030058 PMC5371813 28335562

[5] H. Huang *et al.*, “COVID-19: A call for physical scientists and engineers,” ACS Nano 14(4), 3747–3754 (2020). 10.1021/acsnano.0c02618 32267678

[6] B. Stephens *et al.*, “Microbial exchange via fomites and implications for human health,” Curr. Pollut. Rep. 5(4), 198–213 (2019). 10.1007/s40726-019-00123-6 PMC7149182 34171005

[7] O. Dumas *et al.*, “Association of occupational exposure to disinfectants with incidence of chronic obstructive pulmonary disease among US female nurses,” JAMA Network Open 2(10), e1913563 (2019). 10.1001/jamanetworkopen.2019.13563 31626315 PMC6813668

[8] P. K. Rai *et al.*, “Tackling COVID-19 pandemic through nanocoatings: Confront and exactitude,” Curr. Res. Green Sustainable Chem. 3, 100011 (2020). 10.1016/j.crgsc.2020.100011

[9] A. W. H. Chin *et al.*, “Stability of SARS-CoV-2 in different environmental conditions,” Lancet Microbe 1(1), e10 (2020). 10.1016/s2666-5247(20)30095-1 32835322 PMC7214863

[10] S.-D. Walji and M. G. Aucoin, “A critical evaluation of current protocols for self-sterilizing surfaces designed to reduce viral nosocomial infections,” Am. J. Infect. Control 48, P1255 (2020). 10.1016/j.ajic.2020.02.008 32204920

[11] Z. Sun and K. Ostrikov, “Future antiviral surfaces: Lessons from COVID-19 pandemic,” Sustainable Mater. Technol. 25, e00203 (2020). 10.1016/j.susmat.2020.e00203

[12] C. Weiss *et al.*, “Toward nanotechnology-enabled approaches against the COVID-19 pandemic,” ACS Nano 14(6), 6383–6406 (2020). 10.1021/acsnano.0c03697 32519842

[13] A. Singh *et al.*, “3. Polymer-based antimicrobial coatings as potential biomaterials: From action to application,” in Handbook of Antimicrobial Coatings, edited by A. Tiwari (Elsevier, 2018), pp. 27–61.

[14] E. O. Ogunsona *et al.*, “Engineered nanomaterials for antimicrobial applications: A review,” Appl. Mater. Today 18, 100473 (2020). 10.1016/j.apmt.2019.100473

[15] S. Galdiero, A. Falanga, M. Vitiello, M. Cantisani, V. Marra, and M. Galdiero, “Silver nanoparticles as potential antiviral agents,” Molecules 16(10), 8894–8918 (2011). 10.3390/molecules16108894 22024958 PMC6264685

[16] G. Reina *et al.*, “Hard nanomaterials in time of viral pandemics,” ACS Nano 14, 9364 (2020). 10.1021/acsnano.0c04117 32667191

[17] J. Kemp, J. Edson, and Y. J. Kwon, “Nano-antibiotics: Nanotechnology in fighting against infectious diseases,” in Handbook of Nanobiomedical Research: Fundamentals, Applications and Recent Developments, Applications in Therapy Vol. 2 (World Scientific, 2014), pp. 373–405.

[18] B. Das and S. Patra, “Antimicrobials: Meeting the challenges of antibiotic resistance through nanotechnology,” in Nanostructures for Antimicrobial Therapy (Elsevier, 2017), pp. 1–22.

[19] M. Rai *et al.*, “Metal nanoparticles: The protective nanoshield against virus infection,” Crit. Rev. Microbiol. 42(1), 46–56 (2016). 10.3109/1040841x.2013.879849 24754250

[20] A. Kumar *et al.*, “Silver-nanoparticle-embedded antimicrobial paints based on vegetable oil,” Nat. Mater. 7(3), 236–241 (2008). 10.1038/nmat2099 18204453

[21] A. M. Fayaz *et al.*, “Inactivation of microbial infectiousness by silver nanoparticles-coated condom: A new approach to inhibit HIV- and HSV-transmitted infection,” Int. J. Nanomed. 7, 5007 (2012). 10.2147/IJN.S34973 PMC3459690 23049252

[22] T. V. M. Sreekanth *et al.*, “Ultra-sonication-assisted silver nanoparticles using Panax ginseng root extract and their anti-cancer and antiviral activities,” J. Photochem. Photobiol., B 188, 6–11 (2018). 10.1016/j.jphotobiol.2018.08.013 30176393

[23] Y. Mori *et al.*, “Antiviral activity of silver nanoparticle/chitosan composites against H1N1 influenza A virus,” Nanoscale Res. Lett. 8(1), 93 (2013). 10.1186/1556-276x-8-93 23421446 PMC3606407

[24] Y. Li *et al.*, “Silver nanoparticle based codelivery of oseltamivir to inhibit the activity of the H1N1 influenza virus through ROS-mediated signaling pathways,” ACS Appl. Mater. Interfaces 8(37), 24385–24393 (2016). 10.1021/acsami.6b06613 27588566

[25] T. Du *et al.*, “Antiviral activity of graphene oxide–silver nanocomposites by preventing viral entry and activation of the antiviral innate immune response,” ACS Appl. Bio Mater. 1(5), 1286–1293 (2018). 10.1021/acsabm.8b00154 34996232

[26] Z. Lin *et al.*, “The inhibition of H1N1 influenza virus-induced apoptosis by silver nanoparticles functionalized with zanamivir,” RSC Adv. 7(2), 742–750 (2017). 10.1039/c6ra25010f

[27] A. K. dos Santos Pereira *et al.*, “Synthesis, crystallographic studies, molecular modeling and in vitro biological studies of silver (I) complexes with aminoadamantane ligands,” Polyhedron 173, 114116 (2019). 10.1016/j.poly.2019.114116

[28] L. Chen and J. Liang, “An overview of functional nanoparticles as novel emerging antiviral therapeutic agents,” Mater. Sci. Eng.: C 112, 110924 (2020). 10.1016/j.msec.2020.110924 PMC7195146 32409074

[29] N. Khandelwal *et al.*, “Application of silver nanoparticles in viral inhibition: A new hope for antivirals,” Dig. J. Nanomater. Biostructures 9(1), 175–186 (2014).

[30] J. L. Elechiguerra *et al.*, “Interaction of silver nanoparticles with HIV-1,” J. Nanobiotech. 3(1), 6–10 (2005). 10.1186/1477-3155-3-6 PMC1190212 15987516

[31] H. H. Lara *et al.*, “Mode of antiviral action of silver nanoparticles against HIV-1,” J. Nanobiotech. 8(1), 1–10 (2010). 10.1186/1477-3155-8-1 PMC2818642 20145735

[32] J. Zhou *et al.*, “Progress and perspective of antiviral protective material,” Adv. Fiber Mater. 2, 123–139 (2020). 10.1007/s42765-020-00047-7 PMC7309218 38624352

[33] R. R. Arvizo *et al.*, “Intrinsic therapeutic applications of noble metal nanoparticles: Past, present and future,” Chem. Soc. Rev. 41(7), 2943–2970 (2012). 10.1039/c2cs15355f 22388295 PMC3346960

[34] Q. H. Quach *et al.*, “Size-dependent neutralizing activity of gold nanoparticle-based subunit vaccine against dengue virus,” Acta Biomater. 78, 224–235 (2018). 10.1016/j.actbio.2018.08.011 30099200

[35] H. Sekimukai *et al.*, “Gold nanoparticle‐adjuvanted S protein induces a strong antigen‐specific IgG response against severe acute respiratory syndrome‐related coronavirus infection, but fails to induce protective antibodies and limit eosinophilic infiltration in lungs,” Microbiol. Immunol. 64(1), 33–51 (2020). 10.1111/1348-0421.12754 31692019 PMC7168429

[36] A. Halder *et al.*, “Highly monodispersed gold nanoparticles synthesis and inhibition of herpes simplex virus infections,” Mater. Sci. Eng.: C 89, 413–421 (2018). 10.1016/j.msec.2018.04.005 29752114

[37] M.-C. Bowman *et al.*, “Inhibition of HIV fusion with multivalent gold nanoparticles,” J. Am. Chem. Soc. 130(22), 6896–6897 (2008). 10.1021/ja710321g 18473457 PMC2916654

[38] M. D. Massich *et al.*, “Regulating immune response using polyvalent nucleic acid− gold nanoparticle conjugates,” Mol. Pharmaceutics 6(6), 1934–1940 (2009). 10.1021/mp900172m PMC3241524 19810673

[39] J. J. Skehel and D. C. Wiley, “Receptor binding and membrane fusion in virus entry: The influenza hemagglutinin,” Annu. Rev. Biochem. 69(1), 531–569 (2000). 10.1146/annurev.biochem.69.1.531 10966468

[40] J. Kim *et al.*, “Porous gold nanoparticles for attenuating infectivity of influenza A virus,” J. Nanobiotech. 18(1), 54 (2020). 10.1186/s12951-020-00611-8 PMC7092597 32209114

[41] S. L. Warnes, Z. R. Little, and C. W. Keevil, “Human coronavirus 229E remains infectious on common touch surface materials,” MBio 6(6), e01697 (2015). 10.1128/mbio.01697-15 26556276 PMC4659470

[42] N. Van Doremalen *et al.*, “Aerosol and surface stability of SARS-CoV-2 as compared with SARS-CoV-1,” N. Engl. J. Med. 382(16), 1564–1567 (2020). 10.1056/nejmc2004973 32182409 PMC7121658

[43] R. B. Thurman, C. P. Gerba, and G. Bitton, “The molecular mechanisms of copper and silver ion disinfection of bacteria and viruses,” Crit. Rev. Environ. Control 18(4), 295–315 (1989). 10.1080/10643388909388351

[44] K. Sunada, M. Minoshima, and K. Hashimoto, “Highly efficient antiviral and antibacterial activities of solid-state cuprous compounds,” J. Hazard. Mater. 235-236, 265–270 (2012). 10.1016/j.jhazmat.2012.07.052 22902129

[45] M. Minoshima *et al.*, “Comparison of the antiviral effect of solid-state copper and silver compounds,” J. Hazard. Mater. 312, 1–7 (2016). 10.1016/j.jhazmat.2016.03.023 27015373 PMC7116991

[46] N. Monmaturapoj *et al.*, “Antiviral activity of multifunctional composite based on TiO_2_-modified hydroxyapatite,” Mater. Sci. Eng.: C 92, 96–102 (2018). 10.1016/j.msec.2018.06.045 30184826

[47] H. Irie, Y. Watanabe, and K. Hashimoto, “Nitrogen-concentration dependence on photocatalytic activity of TiO_2−*x*_N_*x*_ powders,” J. Phys. Chem. B 107(23), 5483–5486 (2003). 10.1021/jp030133h

[48] P. V. Kamat and D. Meisel, “Nanoparticles in advanced oxidation processes,” Curr. Opin. Colloid Interface Sci. 7(5), 282–287 (2002). 10.1016/s1359-0294(02)00069-9

[49] M. Anpo *et al.*, “Design of unique titanium oxide photocatalysts by an advanced metal ion-implantation method and photocatalytic reactions under visible light irradiation,” Res. Chem. Intermed. 24(2), 143–149 (1998). 10.1163/156856798x00735

[50] S. T. Martin, C. L. Morrison, and M. R. Hoffmann, “Photochemical mechanism of size-quantized vanadium-doped TiO_2_ particles,” J. Phys. Chem. 98(51), 13695–13704 (1994). 10.1021/j100102a041

[51] A. Di Paola *et al.*, “Preparation of polycrystalline TiO_2_ photocatalysts impregnated with various transition metal ions: Characterization and photocatalytic activity for the degradation of 4-nitrophenol,” J. Phys. Chem. B 106(3), 637–645 (2002). 10.1021/jp013074l

[52] L. Zang *et al.*, “Visible‐light detoxification and charge generation by transition metal chloride modified titania,” Chem.-Eur. J. 6(2), 379–384 (2000). 10.1002/(sici)1521-3765(20000117)6:2<379::aid-chem379>3.0.co;2-z 11931119

[53] M. Liu *et al.*, “Visible-light sensitive Cu(II)–TiO_2_ with sustained anti-viral activity for efficient indoor environmental remediation,” J. Mater. Chem. A 3(33), 17312–17319 (2015). 10.1039/c5ta03756e

[54] K. Kaviyarasu *et al.*, “Photocatalytic performance and antimicrobial activities of HAp-TiO_2_ nanocomposite thin films by sol-gel method,” Surf. Interfaces 6, 247–255 (2017). 10.1016/j.surfin.2016.10.002

[55] S. Ma *et al.*, “Facile fabrication of C–TiO_2_ nanocomposites with enhanced photocatalytic activity for degradation of tetracycline,” ACS Omega 4(25), 21063–21071 (2019). 10.1021/acsomega.9b02411 31867498 PMC6921265

[56] B. Czech *et al.*, “Sorption of pharmaceuticals and personal care products (PPCPs) onto a sustainable cotton based adsorbent,” Sustainable Chem. Pharm. 18, 100324 (2020). 10.1016/j.scp.2020.100324

[57] X. Meng *et al.*, “Hydrothermal preparation of Mn_0.5_Cd_0.5_S/carbon nanotubes nanocomposite photocatalyst with improved H_2_ production performance,” Mater. Res. Bull. 135, 111156 (2021). 10.1016/j.materresbull.2020.111156

[58] V. G. Ahmadabadi *et al.*, “Structure-rate performance relationship in Si nanoparticles-carbon nanofiber composite as flexible anode for lithium-ion batteries,” Electrochim. Acta 330, 135232 (2020). 10.1016/j.electacta.2019.135232

[59] K. Shirvanimoghaddam *et al.*, “Periodical patterning of a fully tailored nanocarbon on CNT for fabrication of thermoplastic composites,” Composites, Part A 107, 304–314 (2018). 10.1016/j.compositesa.2018.01.015

[60] K. Shirvanimoghaddam *et al.*, “Sustainable carbon microtube derived from cotton waste for environmental applications,” Chem. Eng. J. 361, 1605–1616 (2019). 10.1016/j.cej.2018.11.157

[61] S. M. Fakhrhoseini *et al.*, “Ultrafast microwave assisted development of magnetic carbon microtube from cotton waste for wastewater treatment,” Colloids Surf., A 606, 125449 (2020). 10.1016/j.colsurfa.2020.125449

[62] K. Shirvanimoghaddam *et al.*, “The light enhanced removal of bisphenol a from wastewater using cotton waste derived carbon microtubes,” J. Colloid Interface Sci. 539, 425–432 (2019). 10.1016/j.jcis.2018.12.090 30599398

[63] K. Shirvanimoghaddam *et al.*, “Super hard carbon microtubes derived from natural cotton for development of high performance titanium composites,” J. Alloys Compd. 775, 601–616 (2019). 10.1016/j.jallcom.2018.10.121

[64] K. Shirvanimoghaddam *et al.*, “Death by waste: Fashion and textile circular economy case,” Sci. Total Environ. 718, 137317 (2020). 10.1016/j.scitotenv.2020.137317 32088483

[65] Z. Meng and W. Oh, “Photodegradation of organic dye by CoS_2_ and carbon (C_60_, graphene, CNT)/TiO_2_ composite sensitizer,” Chin. J. Catal. 33(9), 1495–1501 (2012). 10.1016/s1872-2067(11)60429-4

[66] K. Shirvanimoghaddam *et al.*, “Thermomechanical performance of cheetah skin carbon nanotube embedded composite: Isothermal and non-isothermal investigation,” Polymer 145, 294–309 (2018). 10.1016/j.polymer.2018.04.079

[67] K. U. Rahman *et al.*, “Flexible bacterial cellulose-based BC–SiO_2_–TiO_2_–Ag membranes with self-cleaning, photocatalytic, antibacterial and UV-shielding properties as a potential multifunctional material for combating infections and environmental applications,” J. Environ. Chem. Eng. 9(1), 104708 (2021). 10.1016/j.jece.2020.104708

[68] H. Samadian *et al.*, “Genotoxicity assessment of carbon-based nanomaterials; Have their unique physicochemical properties made them double-edged swords?,” Mutat. Res., Rev. Mutat. Res. 783, 108296 (2020). 10.1016/j.mrrev.2020.108296 32192648

[69] J. Xu, X. Lu, and B. Li, “Synthesis, functionalization, and characterization,” *Biomedical Applications and Toxicology of Carbon Nanomaterials* (Wiley, 2016), Chap. 1.

[70] K. Shirvanimoghaddam *et al.*, “Cheetah skin structure: A new approach for carbon-nano-patterning of carbon nanotubes,” Composites, Part A 95, 304–314 (2017). 10.1016/j.compositesa.2017.01.023

[71] M. J. Meziani *et al.*, “Visible-light-activated bactericidal functions of carbon ‘Quantum’ dots,” ACS Appl. Mater. Interfaces 8(17), 10761–10766 (2016). 10.1021/acsami.6b01765 27064729 PMC5017886

[72] D. Ting *et al.*, “Multisite inhibitors for enteric coronavirus: Antiviral cationic carbon dots based on curcumin,” ACS Appl. Nano Mater. 1(10), 5451–5459 (2018). 10.1021/acsanm.8b00779 35286056

[73] T. Du *et al.*, “Carbon dots as inhibitors of virus by activation of type I interferon response,” Carbon 110, 278–285 (2016). 10.1016/j.carbon.2016.09.032

[74] X. Dong *et al.*, “Carbon dots’ antiviral functions against noroviruses,” Sci. Rep. 7(1), 519 (2017). 10.1038/s41598-017-00675-x 28364126 PMC5428842

[75] M. Z. Fahmi *et al.*, “Design of boronic acid-attributed carbon dots on inhibits HIV-1 entry,” RSC Adv. 6(95), 92996–93002 (2016). 10.1039/c6ra21062g

[76] Y. Y. Aung *et al.*, “Inactivation of HIV-1 infection through integrative blocking with amino phenylboronic acid attributed carbon dots,” ACS Biomater. Sci. Eng. 6, 4490 (2020). 10.1021/acsbiomaterials.0c00508 33455181

[77] S. H. Friedman *et al.*, “Inhibition of the HIV-1 protease by fullerene derivatives: Model building studies and experimental verification,” J. Am. Chem. Soc. 115(15), 6506–6509 (1993). 10.1021/ja00068a005

[78] R. Sijbesma *et al.*, “Synthesis of a fullerene derivative for the inhibition of HIV enzymes,” J. Am. Chem. Soc. 115(15), 6510–6512 (1993). 10.1021/ja00068a006

[79] J. T. Rhule *et al.*, “Polyoxometalates and fullerenes as anti-HIV agents,” Metallopharmaceuticals II (Springer, 1999), pp. 117–137.

[80] S. Goodarzi *et al.*, “Fullerene: Biomedical engineers get to revisit an old friend,” Mater. Today 20(8), 460–480 (2017). 10.1016/j.mattod.2017.03.017

[81] M. Möritz *et al.*, “Capability of air filters to retain airborne bacteria and molds in heating, ventilating and air-conditioning (HVAC) systems,” Int. J. Hyg. Environ. Health 203(5), 401–409 (2001). 10.1078/1438-4639-00054 11556144

[82] D. Pradhan *et al.*, “A review of current interventions for COVID-19 prevention,” Arch. Med. Res. 51(5), 363–374 (2020). 10.1016/j.arcmed.2020.04.020 32409144 PMC7190516

[83] P. Erkoc and F. Ulucan-Karnak, “Nanotechnology-based antimicrobial andantiviral surface coating strategies,” Prosthesis 3, 25–52 (2021). 10.3390/prosthesis3010005

[84] P. J. M. Brouwer *et al.*, “Potent neutralizing antibodies from COVID-19 patients define multiple targets of vulnerability,” Science 369(6504), 643–650 (2020). 10.1126/science.abc5902 32540902 PMC7299281

[85] A. Tavakoli *et al.*, “Polyethylene glycol-coated zinc oxide nanoparticle: An efficient nanoweapon to fight against herpes simplex virus type 1,” Nanomedicine 13(21), 2675–2690 (2018). 10.2217/nnm-2018-0089 30346253

[86] C. Balagna *et al.*, “Virucidal effect against coronavirus SARS-CoV-2 of a silver nanocluster/silica composite sputtered coating,” Open Ceram. 1, 100006 (2020). 10.1016/j.oceram.2020.100006

[87] X. Hang *et al.*, “Antiviral activity of cuprous oxide nanoparticles against Hepatitis C Virus *in vitro*,” J. Virol. Methods 222, 150–157 (2015). 10.1016/j.jviromet.2015.06.010 26116793

[88] A. Salleh, R. Naomi, N. D. Utami, A. W. Mohammad, E. Mahmoudi, N. Mustafa, and M. B. Fauzi, “The potential of silver nanoparticles for antiviral and antibacterial applications: A mechanism of action,” Nanomater. 10, 1566 (2020). 10.3390/nano10081566 PMC7466543 32784939

[89] Y. Fujimori *et al.*, “Novel antiviral characteristics of nanosized copper(I) Iodide particles showing inactivation activity against 2009 pandemic H1N1 influenza virus,” Appl. Environ. Microbiol. 78(4), 951–955 (2012). 10.1128/aem.06284-11 22156433 PMC3272987

[90] V. Lysenko *et al.*, “Nanoparticles as antiviral agents against adenoviruses,” Adv. Nat. Sci.: Nanosci. Nanotechnol. 9(2), 025021 (2018). 10.1088/2043-6254/aac42a

[91] R. Vazquez-Munoz and J. L. Lopez-Ribot, “Nanotechnology as an alternative to reduce the spread of COVID-19,” Challenges 11(2), 15 (2020). 10.3390/challe11020015

[92] D. Morris *et al.*, “Antiviral and immunomodulatory activity of silver nanoparticles in experimental RSV infection,” Viruses 11(8), 732 (2019). 10.3390/v11080732 PMC6723559 31398832

[93] V. Cagno *et al.*, “Broad-spectrum non-toxic antiviral nanoparticles with a virucidal inhibition mechanism,” Nat. Mater. 17(2), 195–203 (2018). 10.1038/nmat5053 29251725

[94] See https://phys.org/news/2020-05-anti-covid-nanocoating-surface.html for more information about their scientific investigations.

[95] See https://www.coatingsworld.com/content-microsite/cw_covid-19/2020-04-15/anti-viral-surface-coating-to-prevent-spread-of-novel-coronavirus-covid-19-through-touch for more information about their scientific investigations.

[96] L. Cao and J. Lu, “Atomic-scale engineering of metal–oxide interfaces for advanced catalysis using atomic layer deposition,” Catal. Sci. Technol. 10(9), 2695–2710 (2020). 10.1039/d0cy00304b

[97] R. Kumar *et al.*, “Antimicrobial properties of ZnO nanomaterials: A review,” Ceram. Int. 43(5), 3940–3961 (2017). 10.1016/j.ceramint.2016.12.062

[98] A. Sirelkhatim *et al.*, “Review on zinc oxide nanoparticles: Antibacterial activity and toxicity mechanism,” Nano-Micro Lett. 7(3), 219–242 (2015). 10.1007/s40820-015-0040-x PMC6223899 30464967

[99] H. Agarwal, S. Venkat Kumar, and S. Rajeshkumar, “A review on green synthesis of zinc oxide nanoparticles—An eco-friendly approach,” Resour.-Effic. Technol. 3(4), 406–413 (2017). 10.1016/j.reffit.2017.03.002

[100] M. Shah, D. Fawcett, S. Sharma, S. Tripathy, and G. Poinern, “Green synthesis of metallic nanoparticles via biological entities,” Materials 8, 7278–7308 (2015). 10.3390/ma8115377 28793638 PMC5458933

[101] S. Zhuiykov, M. K. Akbari, Z. Hai, C. Xue, H. Xu, and L. Hyde, “Wafer-scale fabrication of conformal atomic-layered TiO_2_ by atomic layer deposition using tetrakis (dimethylamino) titanium and H_2_O precursors,” Mater. Des. 120, 99–108 (2017). 10.1016/j.matdes.2017.02.016 PMC5480828 28664177

[102] H. Xu *et al.*, “Ultra-thin MoO_3_ film goes wafer-scaled nano-architectonics by atomic layer deposition,” Mater. Des. 149, 135–144 (2018). 10.1016/j.matdes.2018.04.007

[103] C. López de Dicastillo *et al.*, “Antimicrobial bilayer nanocomposites based on the incorporation of as-synthetized hollow zinc oxide nanotubes,” Nanomaterials 10(3), 503 (2020). 10.3390/nano10030503 PMC7153247 32168893

[104] M. Naebe and K. Shirvanimoghaddam, “Functionally graded materials: A review of fabrication and properties,” Appl. Mater. Today 5, 223–245 (2016). 10.1016/j.apmt.2016.10.001

[105] L. A. Ikner *et al.*, “A continuously active antimicrobial coating effective against human coronavirus 229E,” medRxiv:2020.05.10.20097329 (2020).

[106] See https://www.ust.hk/news/research-and-innovation/hkust-develops-new-smart-anti-microbial-coating-fight-against-covid-19 for more information about their scientific investigations.

[107] See https://www.railtech.com/coronavirus/2020/03/23/disinfecting-trains-with-titanium-dioxide/?gdpr=accept for more information about their scientific investigations.

[108] D. P. Otto and M. M. de Villiers, “Layer-by-layer nanocoating of antiviral polysaccharides on surfaces to prevent coronavirus infections,” Molecules 25(15), 3415 (2020). 10.3390/molecules25153415 PMC7435837 32731428

[109] A. Elzaabalawy, P. Verberne, and S. A. Meguid, “Multifunctional silica–silicone nanocomposite with regenerative superhydrophobic capabilities,” ACS Appl. Mater. Interfaces 11(45), 42827–42837 (2019). 10.1021/acsami.9b15445 31623429

[110] S. A. Meguid and A. Elzaabalawy, “Potential of combating transmission of COVID-19 using novel self-cleaning superhydrophobic surfaces: Part I—Protection strategies against fomites,” Int. J. Mech. Mater. Des. 16(3), 423–431 (2020). 10.1007/s10999-020-09513-x PMC7405757 38624551

[111] V. Palmieri *et al.*, “Bacteria meet graphene: Modulation of graphene oxide nanosheet interaction with human pathogens for effective antimicrobial therapy,” ACS Biomater. Sci. Eng. 3(4), 619–627 (2017). 10.1021/acsbiomaterials.6b00812 33429629

[112] V. Palmieri *et al.*, “Graphene oxide touches blood: In vivo interactions of bio-coronated 2D materials,” Nanoscale Horiz. 4(2), 273–290 (2019). 10.1039/c8nh00318a 32254085

[113] O. Akhavan, M. Choobtashani, and E. Ghaderi, “Protein degradation and RNA efflux of viruses photocatalyzed by graphene–tungsten oxide composite under visible light irradiation,” J. Phys. Chem. C 116(17), 9653–9659 (2012). 10.1021/jp301707m

[114] B. Ziem *et al.*, “Size-dependent inhibition of herpesvirus cellular entry by polyvalent nanoarchitectures,” Nanoscale 9(11), 3774–3783 (2017). 10.1039/c7nr00611j 28266670

[115] V. Palmieri *et al.*, “The future development of bacteria fighting medical devices: The role of graphene oxide,” Expert Rev. Med. Devices 13(11), 1013–1019 (2016). 10.1080/17434440.2016.1245612 27710143

[116] Z. Song *et al.*, “Virus capture and destruction by label-free graphene oxide for detection and disinfection applications,” Small 11(9-10), 1171–1176 (2015). 10.1002/smll.201401706 25285820

[117] D. Iannazzo *et al.*, “Graphene quantum dots based systems as HIV inhibitors,” Bioconjugate Chem. 29(9), 3084–3093 (2018). 10.1021/acs.bioconjchem.8b00448 30106563

[118] X. Du *et al.*, “Hypericin-loaded graphene oxide protects ducks against a novel duck reovirus,” Mater. Sci. Eng.: C 105, 110052 (2019). 10.1016/j.msec.2019.110052 31546360

[119] M. G. Stanford *et al.*, “Self-Sterilizing laser-induced graphene bacterial air filter,” ACS Nano 13(10), 11912–11920 (2019). 10.1021/acsnano.9b05983 31560513

[120] L. Yip *et al.*, “Influenza virus RNA recovered from droplets and droplet nuclei emitted by adults in an acute care setting,” J. Occup. Environ. Hyg. 16(5), 341–348 (2019). 10.1080/15459624.2019.1591626 31050610 PMC7157967

[121] V. Palmieri and M. Papi, “Can graphene take part in the fight against COVID-19?,” Nano Today 33, 100883 (2020). 10.1016/j.nantod.2020.100883 32382315 PMC7203038

[122] Y. Li *et al.*, “Antimicrobial effect of surgical masks coated with nanoparticles,” J. Hosp. Infect. 62(1), 58–63 (2006). 10.1016/j.jhin.2005.04.015 16099072

[123] Z. Lin *et al.*, “Superhydrophobic, photo-sterilize, and reusable mask based on graphene nanosheet-embedded carbon (GNEC) film,” Nano Res. 14(4), 1110–1115 (2021). 10.1007/s12274-020-3158-1 PMC7685909 33250970

[124] H. Zhong *et al.*, “Reusable and recyclable graphene masks with outstanding superhydrophobic and photothermal performances,” ACS Nano 14(5), 6213–6221 (2020). 10.1021/acsnano.0c02250 32329600

